# Delivery room resuscitation and short-term outcomes in very preterm infants: a multicenter cross-sectional study in China

**DOI:** 10.3389/fped.2024.1438780

**Published:** 2024-10-02

**Authors:** Hanni Lin, Zhangbin Yu, Jinjie Huang, Ting Yang, Shitao Duan, Yanping Guo, Shujuan Zeng, Ping Jiang, Rui Wang, Jing Zhang, Lu Ding, Jiebo Liu

**Affiliations:** ^1^ Department of Neonatology, Shenzhen Luohu People’s Hospital, Shenzhen, Guangdong, China; ^2^ Department of Neonatology, Shenzhen People’s Hospital, Shenzhen, Guangdong, China; ^3^ Department of Neonatology, Shenzhen Baoan Women’s and Children’s Hospital, Jinan University, Shenzhen, Guangdong, China; ^4^ Department of Neonatology, Longgang District Maternity & Child Healthcare Hospital of Shenzhen City (Longgang Maternity and Child Institute of Shantou University Medical College), Shenzhen, Guangdong, China; ^5^ Department of Pediatrics, Peking University Shenzhen Hospital, Shenzhen, Guangdong, China; ^6^ Department of Neonatology, Longgang District Central Hospital of Shenzhen, Shenzhen, Guangdong, China

**Keywords:** resuscitation, delivery room, preterm, infant, gestational age

## Abstract

**Objective:**

To explore the risk factors of delivery room (DR) resuscitation and assess the association of DR resuscitation with neonatal outcomes in very preterm infants (VPIs).

**Methods:**

A multicenter retrospective cross-sectional study included VPIs with gestational age (GA) <32 weeks born between January, 2022 and June, 2023 and admitted to neonatal intensive care units of six tertiary hospitals in Shenzhen within 24 h after birth. They were divided into routine care group, positive-pressure ventilation (PPV) group, and endotracheal intubation (ETT) group based on the highest intensity of resuscitation received at birth. The association of antepartum and intrapartum risk factors and short-term outcomes with the intensity of DR resuscitation was evaluated.

**Results:**

Of 683 infants included in this study, 170 (24.9%) received routine care, 260 (38.1%) received bag and mask ventilation or T-piece ventilation and 253 (37%) received ETT. Among the antepartum and intrapartum factors, exposure to antenatal steroids (ANS) decreased the likelihood of ETT. Increasing GA decreased the likelihood of receiving a higher level of DR resuscitation. Among the neonatal outcomes, increasing intensity of DR resuscitation was associated with a raise in the risk of Bronchopulmonary dysplasia. Higher levels of DR resuscitation were associated with the risk of early-onset sepsis. ETT was significantly associated with an increased risk of death.

**Conclusion:**

Among VPIs, low GA and no ANS use increased the risk of high-intensity DR resuscitation interventions; and those who receiving ETT were associated with an increased risk of adverse clinical outcomes.

## Introduction

With the rapid development of perinatal medicine and the continuous promotion of neonatal resuscitation training programs, advances in medical technology have increased the willingness and ability to save premature infants, which has correspondingly led to an increase in the incidence of preterm births worldwide, from 9.8% in 2000 to 10.6% in 2014 (1). In China, the overall preterm birth rate increased from 5.9% in 2012 to 6.4% in 2018, and the very preterm birth rate increased from 0.6% in 2012 to 0.7% in 2018 (2), and increasingly younger gestational ages (GAs) of preterm infants are being treated.

As the GA of preterm infants decreases, the more immature their anatomical and physiological characteristics become, resulting in the inability to establish spontaneous respiration or ineffective spontaneous respiration at birth and the need for resuscitative interventions to help establish effective spontaneous respiration to transition from the intrauterine environment to the extrauterine environment. Fischer et al. reported that 6%–7% of very preterm infants (VPIs) were unable to successfully transition to the extrauterine environment in the early stages of life and required the extensive delivery room (DR) cardiopulmonary resuscitation, defined as chest compressions with or without epinephrine (3).

A standardized set of neonatal resuscitation practices was published jointly by the American Heart Association and the American Academy of Pediatrics in 2021, including the initial resuscitation steps, positive pressure ventilation (PPV), endotracheal intubation (ETT), chest compressions, and medications (4). Most preterm infants are stabilized by noninvasive respiratory support after birth, but a multicenter retrospective cohort study published in 2022 showed that 41.9% of preterm infants less than 32 weeks of GA required invasive respiratory support by ETT for stabilization, and 10.3% of preterm infants experienced failure with initial continuous positive airway pressure (CPAP) support (5). Therefore, the initial stabilization of preterm infants after birth is of great importance.

Available studies have shown that higher intensity of resuscitation, such as DR intubation and cardiopulmonary resuscitation, is associated with increased mortality in preterm infants and incidence of serious complications such as bronchopulmonary dysplasia (BPD) and neurological impairment (3, 6–8). Evidence suggests that antenatal steroids (ANS) and intrapartum resuscitation strategies may reduce the need for ETT (9–11), so perinatal management and resuscitation strategies adopted at delivery can greatly influence the clinical outcomes.

In China, there are unified and updated guidelines for neonatal resuscitation (12), but resuscitation practices still vary among medical centers. This study retrospectively collected clinical data on VPIs with GA <32 weeks after resuscitation in six tertiary NICUs in Shenzhen, aiming to study the association between antepartum and intrapartum factors and the extent of DR resuscitation and the short-term outcomes of VPIs after DR resuscitation.

## Methods

### Study population

A cross-sectional observational study retrospectively analyzed VPIs born between January 1, 2022 and June 30, 2023 with GA <32 weeks and admitted to the neonatal intensive care units (NICUs) of six tertiary hospitals in Shenzhen (four tertiary general hospitals and two tertiary maternity and child healthcare hospitals) within 24 h after birth. Data on readmissions and transfers between participating hospitals considered to be the same infant were tracked. Infants were followed until NICU discharge or transfer or death. Infants refused resuscitation by parents at birth, admitted to the NICU after 24 h of birth, with incomplete clinical data, and transferred to non-participating hospitals within 24 h of hospitalization were excluded. Shenzhen People's Hospital Ethics Committee approved this study (approval no. LL-KY-2022288).

The enrolled infants were categorized into three groups according to the highest intensity of DR resuscitation they received at birth: routine care, PPV and ETT. Routine care was defined as warmth, suction if necessary and mild stimulation. PPV was defined as ventilation via bag and mask or T-piece. ETT was defined as PPV via ETT.

### Data collection

Data on the following variables were extracted from data deposited in the Shenzhen Neonatal Data Network (SNDN) database, which was established by SNDN with the primary goal of conducting high-quality collaborative research dedicated to improving neonatal-perinatal health in Shenzhen, China (13), and launched with retrospective data collection starting from January 1, 2022 for the infants who met the study criteria:

1. Maternal information: age, number of deliveries, antenatal care, diabetes, hypertension, anemia in pregnancy, intrahepatic cholestasis in pregnancy, chorioamnionitis, antenatal hemorrhage, placenta previa, placental abruptio placenta, premature rupture of membranes, use of ANS, use of antenatal magnesium sulfate, mode of conception, and mode of delivery.
2. Neonatal information: GA, small for gestational age, birth weight, sex, multiple births, 1-min Apgar score, 5-min Apgar score, and birth resuscitation measures.
3. Neonatal outcome: respiratory distress syndrome (RDS), BPD, early-onset sepsis (EOS), late-onset sepsis (LOS), intracranial hemorrhage (ICH), cystic periventricular leukomalacia (cPVL), necrotizing enterocolitis (NEC), retinopathy of prematurity (ROP) and death.

Trained data abstractors were responsible for data acquisition in each hospital. Data were directly entered into the Perinatal Cloud Database (https://www.perinatalcloud.com/) customized by SNDN, which collects data including maternal information, neonatal information, antenatal care, major morbidities, and outcomes at discharge. The database has built-in error-checking functionality with a standard manual of operations and definitions. To ensure the confidentiality of infant identity, each hospital and its patients were assigned a unique identification number. Each hospital designated an attending (or above) neonatologist as the principal investigator, who was trained and assessed by the SNDN coordinating center and was responsible for data quality control at each hospital after passing the assessment.

### Definitions

Chorioamnionitis: acute histologic changes on examination of the placenta and/or clinical signs such as fever, uterine fundal tenderness, maternal tachycardia (>100/min), fetal tachycardia (>160/min) and purulent or foul-smelling amniotic fluid. ANS: the mother had received at least one dose of dexamethasone intravenously or intramuscularly at any time before delivery. BPD: continuous oxygen use for ≥28 days after birth, GA <32 weeks assessed at postmenstrual age of 36 weeks or at discharge, and GA ≥32 weeks assessed at 56 days postnatal age or at discharge, no oxygen use was considered mild; need for oxygen with Fraction of inspiration O₂ (FiO2) <30% was considered moderate; and FiO2 ≥30% or the need for CPAP, mechanical ventilation is considered severe (14). cPVL: presence of periventricular cysts on cranial ultrasound or magnetic resonance imaging (15). Early death: death within 7 days of birth.

The diagnostic criteria of RDS, EOS, LOS, ICH, NEC and ROP were according to the textbook Practice of Neonatology (5th Edition) (16).

### Statistical analysis

SPSS, version 27.0 (IBMCorp., Armonk, N.Y., USA), was used for all the statistical analysis. According to distribution, continuous variables were described as median and interquartile range (IQR) [median (IQR)] and comparison among groups were performed using Kruskal-Wallis *H*-test. Categorical variables were described as frequency and rate [*n* (%)] and comparison among groups were performed using χ^2^ test or Fisher's exact test. Multivariate logistic regression analysis was used in the multivariate study to explore the risk factors of DR resuscitation and to assess the association of DR resuscitation and short-term outcomes, adjusting for maternal age, birth weight, and cesarean section. *P* < 0.05 was considered statistically significant.

## Results

### Study population

**Figure 1:**
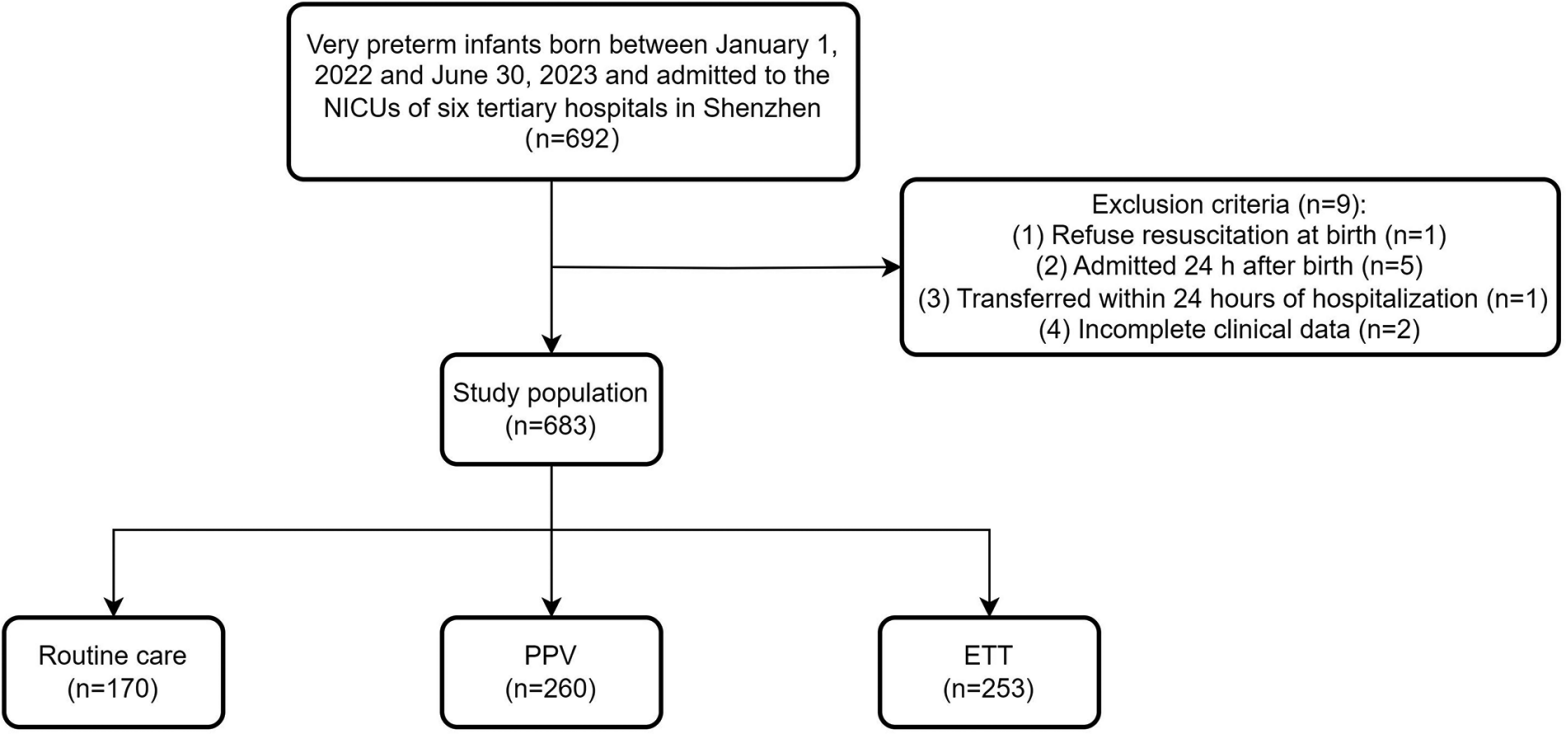
Flow chart for the study population. NICU, neonatal intensive care unit; PPV, positive pressure ventilation; ETT, endotracheal intubation.

A total of 692 VPIs were born between January 1, 2022 and June 30, 2023 and admitted to the NICUs of six tertiary hospitals in Shenzhen, one infant with parental refusal of DR resuscitation, five infants with admission 24 h after birth, two infants with incomplete clinical data, one infant transferred to non-participating hospitals within 24 h of hospitalization were excluded from this analysis. 683 infants met the enrollment criteria, 170 (24.9%) received routine care without additional resuscitation; 260 (38.1%) received PPV; and 253 (37%) received ETT (Figure 1).

### Variation across gestational age

**Figure 2:**
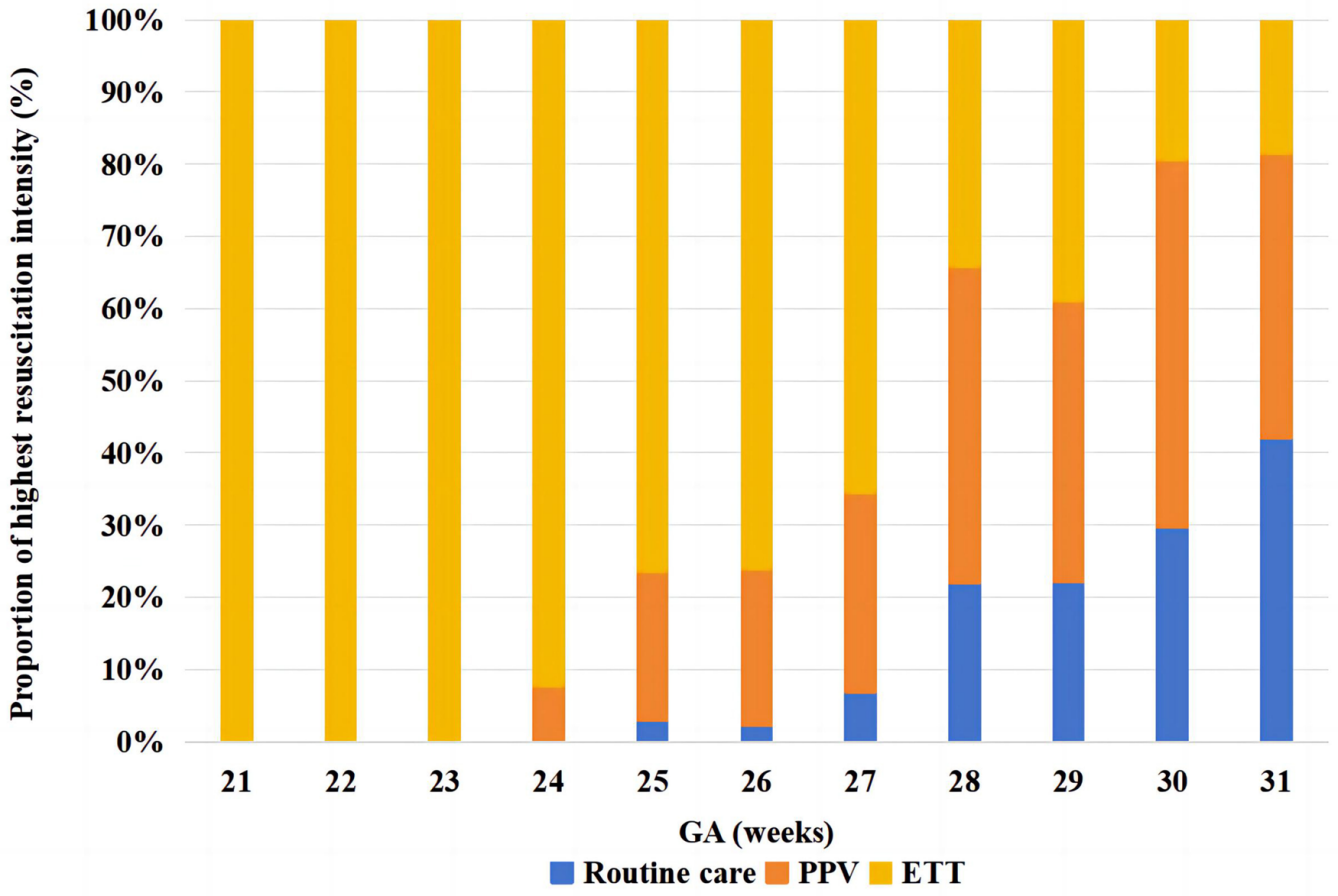
Variation in delivery room resuscitation intensity by gestational age in very preterm infants. The X-axis represents GA, and the Y-axis represents the proportion of each intensity of resuscitation. Different colors represent different resuscitation intensities. GA, gestational age; PPV, positive pressure ventilation; ETT, endotracheal intubation.

The proportion of resuscitation intensity received varied by GA in VPIs (Figure 2). The range of GA of enrolled VPIs was 22^+5^–31^+6^ weeks, the median GA was 30 weeks (IQR, 28.0–31.0). All infants born at less than 24 weeks of GA received ETT at birth, whereas less than 25% of infants born at more than 29 weeks of GA received ETT. As the GA increasing, the proportion of infants receiving ETT gradually declined, although there was a slight decrease in the proportion of infants receiving ETT at 29 weeks' GA compared to those at 28 weeks' GA, but the difference was not statistically significant.

### Baseline characteristics for DR resuscitation in VPIs

Maternal and neonatal characteristics are shown in Table 1. Maternal age was elevated in the ETT group compared to the routine care group (*P* < 0.05). Maternal chorioamnionitis and cesarean section were associated with intensity of resuscitation (*P* < 0.05). Lack of ANS, lower birth weight and GA, As the intensity of resuscitation increased, the Apgar scores at 1 min and 5 min gradually decreased (*P* < 0.05). The logistic regression model exploring the relationship of maternal and neonatal characteristics with different levels of DR resuscitation adjusted for clinically important risk factors such as maternal age, caesarean section and birth weight (Table 2). Compared with the routine care group, maternal chorioamnionitis reduced the odds of PPV (aOR = 0.650; 95% CI = 0.429–0.983), exposure to ANS (aOR = 0.251; 95% CI = 0.105–0.602) reduced the odd of ETT, and increasing GA decreased the likelihoods of PPV (aOR = 0.716; 95% CI = 0.592–0.865) and ETT (aOR = 0.582; 95% CI = 0.475–0.713).

**Table 1:** Baseline characteristics of three groups in very preterm infants.

Baseline characteristics	Routine care (*n* = 170)	PPV (*n* = 260)	ETT (*n* = 253)	*P*-value
Maternal characteristics				
Maternal age (years), median (IQR)	31 (28,35)	31 (28,34)	32 (29,36)	0.043
Primigravida, *n* (%)	95 (55.9%)	147 (56.5%)	132 (52.2%)	0.577
Antenatal care, *n* (%)	152 (89.4%)	239 (91.9%)	233 (92.1%)	0.578
Maternal diabetes, *n* (%)	56 (32.9%)	73 (28.1%)	68 (26.9%)	0.379
Maternal hypertension, *n* (%)	33 (19.4%)	54 (20.8%)	49 (19.4%)	0.908
Anemia in pregnancy, *n* (%)	25 (14.7%)	53 (20.4%)	35 (13.8%)	0.103
Intrahepatic cholestasis in pregnancy, *n* (%)	5 (2.9%)	10 (3.9%)	5 (2%)	0.446
Chorioamnionitis, *n* (%)	91 (53.5%)	116 (44.6%)	141 (55.7%)	0.031
Antenatal haemorrhage, *n* (%)	14 (8.2%)	27 (10.4%)	22 (8.7%)	0.704
Placenta praevia, *n* (%)	2 (1.2%)	9 (3.5%)	7 (2.8%)	0.347
Placental abruptio placenta, *n* (%)	7 (4.1%)	8 (3.1%)	5 (2%)	0.433
PROM ≥18 h, *n* (%)	39 (22.9%)	63 (24.2%)	46 (18.2%)	0.225
ANS, *n* (%)	162 (95.3%)	234 (90%)	215 (85%)	0.003
Antenatal magnesium sulfate, *n* (%)	130 (76.5%)	204 (78.5%)	188 (74.3%)	0.541
ART, *n* (%)	39 (22.9%)	75 (28.8%)	66 (26.1%)	0.394
Cervical cerclage, *n* (%)	8 (4.7%)	26 (10%)	29 (11.5%)	0.054
Cesarean section, *n* (%)	112 (65.9%)	196 (75.4%)	158 (62.5%)	0.005
Neonatal characteristics				
GA (weeks), median (IQR)	30.79 (29.68, 31.57)	30.29 (28.57, 31.14)	28 (26.29, 30.07)	<0.001
SGA, *n* (%)	7 (4.1%)	7 (2.7%)	15 (5.9%)	0.191
Birth weight (g), median (IQR)	1,435 (1,225, 1,600)	1,300 (1,100, 1,550)	1,010 (760, 1,290)	<0.001
Male, *n* (%)	89 (52.40%)	147 (56.5%)	144 (56.9%)	0.608
Multiple birth, *n* (%)	70 (41.2%)	106 (40.8%)	91 (36%)	0.437
1-min Apgar, median (IQR)	10 (9, 10)	9 (8, 9)	8 (5, 8)	<0.001
5-min Apgar, median (IQR)	10 (10, 10)	10 (9, 10)	9 (8, 10)	<0.001

PPV, positive pressure ventilation; ETT, endotracheal intubation; PROM, premature rupture of membranes; ANS, antenatal steroid; ART, assisted reproductive technology; GA, gestational age; SGA, small for gestational age.

**Table 2:** Estimates of crude odds ratios (cOR) and adjusted odds ratios (aOR) of baseline characteristics for three groups in very preterm infants.

Baseline characteristics	PPV vs. Routine care		ETT vs. Routine care		ETT vs. PPV	
	cOR (95% CI)	aOR (95% CI)	cOR (95% CI)	aOR (95% CI)	cOR (95% CI)	aOR (95% CI)
Chorioamnionitis	0.605 (0.403, 0.907)*	0.650 (0.429, 0.983)*	0.708 (0.450, 1.115)	0.804 (0.505, 1.279)	1.171 (0.796, 1.722)	1.237 (0.834, 1.834)
ANS	0.451 (0.197, 1.033)	0.444 (0.192, 1.027)	0.268 (0.113, 0.635)*	0.251 (0.105, 0.602)*	0.595 (0.328, 1.077)	0.565 (0.306, 1.041)
GA	0.722 (0.626, 0.833)*	0.716 (0.592, 0.865)*	0.471 (0.406, 0.547)*	0.582 (0.475, 0.713)*	0.652 (0.589, 0.722)*	0.813 (0.690, 0.957)

Logistic regression analysis controlled for maternal age, cesarean section and birth weight. PPV, positive pressure ventilation; ETT, endotracheal intubation; ANS, antenatal steroid; GA, gestational age.

* *P* < 0.05.

### Short-term outcomes with different levels of DR resuscitation in VPIs

Short-term outcomes among VPIs with different levels of resuscitation are noted in Table 3. The rates of adverse outcomes including RDS, BPD, EOS, ICH, ROP, and death increased with the increasing level of DR resuscitation (*P* < 0.05). Logistic regression analysis after adjusting for clinically important risk factors (maternal age, cesarean section, and birth weight) showed that the rate of BPD increased as the intensity of DR resuscitation increased, with the highest rate of BPD in the ETT group of VPIs, and increasing intensity of DR resuscitation was associated with a raise in the risk of BPD. Compared with the routine care group, the risk of EOS were higher for VPIs in both the PPV group (aOR = 2.976; 95% CI = 1.186–7.465) and the ETT group (aOR = 3.024; 95% CI = 1.172–7.803). Higher levels of DR resuscitation were associated with the risk of EOS. The risk of death for VPIs was significantly increased in the ETT group compared with the routine care group and the PPV group (Table 4).

**Table 3:** Short-term outcomes of three groups in very preterm infants.

Short-term outcomes	Routine care (*n* = 170)	PPV (*n* = 260)	ETT (*n* = 253)	*P*-value
Pneumothorax, *n* (%)	2 (1.2%)	6 (2.3%)	4 (1.6%)	0.759*
RDS, *n* (%)	119 (70%)	188 (72.3%)	215 (85%)	<0.001
BPD, *n* (%)	30 (17.6%)	81 (31.2%)	137 (54.2%)	<0.001
Mild	25 (14.7%)	51 (19.6%)	75 (29.6%)	
Moderate to severe	5 (2.9%)	30 (11.5%)	62 (24.5%)	
EOS, *n* (%)	6 (3.5%)	29 (11.2%)	43 (17%)	<0.001
LOS, *n* (%)	6 (3.5%)	10 (3.8%)	16 (6.3%)	0.295
ICH, *n* (%)	17 (10%)	37 (14.2%)	70 (27.7%)	<0.001
cPVL, *n* (%)	3 (1.8%)	3 (1.2%)	6 (2.4%)	0.576
≥2 stage NEC, *n* (%)	5 (2.9%)	6 (2.3%)	12 (4.7%)	0.292
ROP, *n* (%)	21 (12.4%)	53 (20.5%)	83 (32.8%)	<0.001
Death, *n* (%)	2 (1.2%)	7 (2.7%)	40 (15.8%)	<0.001
Early death, *n* (%)	1 (0.6%)	4 (1.5%)	26 (10.3%)	

PPV, positive pressure ventilation; ETT, endotracheal intubation; RDS, respiratory distress syndrome; BPD, bronchopulmonary dysplasia; EOS, early-onset sepsis; LOS, late-onset sepsis; ICH, intracranial hemorrhage; cPVL, cystic periventricular leukomalacia; NEC, necrotizing enterocolitis; ROP, retinopathy of prematurity.

* Fisher exact test.

**Table 4:** Estimates of crude odds ratios (cOR) and adjusted odds ratios (aOR) of short-term outcomes for three groups in very preterm infants.

Short-term outcomes	PPV vs. Routine care		ETT vs. Routine care		ETT vs. PPV	
	cOR (95% CI)	aOR (95% CI)	cOR (95% CI)	aOR (95% CI)	cOR (95% CI)	aOR (95% CI)
RDS	0.968 (0.625, 1.501)	0.938 (0.601, 1.462)	1.637 (0.967, 2.771)	1.392 (0.811, 2.387)	1.69 (1.053, 2.713)*	1.484 (0.914, 2.411)
BPD	1.891 (1.153, 3.103)*	1.727 (1.019, 2.926)*	4.94 (2.979, 8.191)*	2.993 (1.738, 5.153)*	2.612 (1.745, 3.911)*	1.733 (1.12, 2.682)*
EOS	2.921 (1.172, 7.28)*	2.976 (1.186, 7.465)*	3.27 (1.289, 8.296)*	3.024 (1.172, 7.803)*	1.12 (0.642, 1.952)	1.016 (0.572, 1.805)
ICH	1.224 (0.654, 2.289)	1.27 (0.67, 2.409)	2.139 (1.142, 4.005)*	1.848 (0.967, 3.533)	1.748 (1.082, 2.824)*	1.455 (0.885, 2.39)
ROP	1.434 (0.807, 2.547)	1.27 (0.703, 2.294)	2.193 (1.226, 3.922)*	1.322 (0.711, 2.458)	1.529 (0.979, 2.39)	1.041 (0.642, 1.689)
Death	2.604 (0.53, 12.802)	1.945 (0.376, 10.064)	27.274 (6.33, 117.517)*	7.988 (1.718, 37.153)*	10.474 (4.442, 24.7)*	4.107 (1.59, 10.608)*

Logistic regression analysis controlled for maternal age, cesarean section and birth weight. PPV, positive pressure ventilation; ETT, endotracheal intubation; RDS, respiratory distress syndrome; BPD, bronchopulmonary dysplasia; EOS, early-onset sepsis; ICH, intracranial hemorrhage; ROP, retinopathy of prematurity.

* *P* < 0.05.

## Discussion

Preterm infants with a GA of less than 32 weeks are in a high-risk group, and most of them need to be resuscitated by healthcare professionals after birth to achieve the transition from fetus to newborn (17). A previous study by Lee et al. showed that lower GA, lower birth weight, abnormal amniotic fluid volume, and no ANS use were associated with resuscitation at birth, especially extensive resuscitation (18). In this study, GA and ANS use of infants in the ETT group were lower than those in the routine care group, which is consistent with previous reported studies (3, 6). The lower GA preterm infants have lower lung maturity and insufficient pulmonary surfactant, which can lead to respiratory insufficiency and increase the possibility of resuscitation; ANS promotes the maturation of fetal lungs, reduces the mortality and the incidence of RDS, NEC, and intraventricular hemorrhage, and improves clinical outcome of preterm infants (19). The Apgar scores at 1 min and 5 min were significantly lower in the ETT group compared to the routine care group, consistent with a report from a multicenter study in China in 2021 (20). 5-min Apgar score possibly reflect the degree of perinatal asphyxia (21), suggesting that infants requiring resuscitation by ETT tend to suffer from perinatal hypoxia, which is one of the reasons why higher intensity resuscitation measures are required in resuscitation. Enhanced perinatal management to reduce the incidence of preterm labor, ANS use in pregnant women with an expectation of preterm labor, identification of high-risk pregnancies, and advance preparation for resuscitation may help to avoid high-intensity resuscitation.

Interestingly, our study found a lower incidence of infants with maternal chorioamnionitis in the PPV group than in the routine care group. A previous study by van Well GTJ et al. (22) had shown that maternal chorioamnionitis stimulates interleukin-6 production by fetal and placental cells, and interleukin-6 promotes lung maturation by stimulating the synthesis of surfactant-associated protein-A, which may reduce the incidence of PPV resuscitation at birth at a certain extent. However, our data showed that the incidence of maternal chorioamnionitis was also high in the ETT group. A study from Kim SY et al. (23) had shown that pro-inflammatory cytokines produced in a response to chorioamnionitis can be aspired by the fetus and make direct contact with respiratory epithelium, thereby inducing pulmonary inflammation. The high incidence of infants with maternal chorioamnionitis in the ETT group did not exclude other risk factors such as coexisting pulmonary infections. Further studies are needed in the future to address the important question of the relationship between maternal chorioamnionitis and the intensity of resuscitation.

The mortality rate of preterm infants in our study was 7.2%, which was similar to the 7.4% reported by the Canadian Neonatal Network (CNN) in 2018 (24), and lower than the 11.8% of data from the China Neonatal Network (CHNN) with 57 tertiary care hospitals in 25 provinces nationwide in 2019 (6). The data for our study came from six tertiary hospitals in the Shenzhen area, and the consideration may be related to Shenzhen is a more economically developed area in China with more developed medical resources. In addition, as the intensity of resuscitation increased, the mortality rate of preterm infants rised, and preterm infants who underwent ETT had the highest mortality rate, especially in the first 7 days after birth, which is consistent with previous reports (6, 20, 25). This suggests that preterm infants undergoing ETT are high-risk infants and need to be focused on and monitored clinically. As clinicians, we need to focus on the changes in the condition of preterm infants resuscitated with ETT for the first 7 days after birth, and closely monitor the vital signs to avoid deterioration of the condition, which may ultimately lead to the occurrence of death.

The present study showed that infants with higher intensity of resuscitation had a higher risk of BPD, and high-intensity resuscitation was an independent risk factor for BPD, which is in accordance with the report of Yoon SJ et al. (25) BPD is not only a common chronic lung disease in preterm infants, but also a systemic disease. Infants with BPD are more likely to develop chronic respiratory disease, cardiovascular disease, and physical and neurological developmental delay (26–31), which imposes a heavy burden on the affected infant, their families, and society. Therefore, it is important to optimize the management strategies for BPD-susceptible infants which can contribute to their health and improve their quality of life.

Wang et al. reported (6) that sepsis is highly correlated with ETT, and our study showed that the rates of EOS and LOS in the ETT group were higher than in the other resuscitation groups, even though there was no difference in the rate of LOS in the different resuscitation intensity groups. The correlation between EOS and higher resuscitation intensities is considered to be due to the fact that EOS is predominantly caused by intrauterine infections, and most preterm infants with intrauterine infections are unable to establish spontaneous respiration well in the early stages of life, and resuscitation is required for a smooth transition.

The strengths of this study include: first, it is a multicenter study on resuscitation of VPIs in Shenzhen, which provides baseline information for quality improvement of resuscitation in preterm infant; second, data abstractors were trained, and strict quality control was applied to the data collected; and third, we found possible differences in resuscitation practices among hospitals in Shenzhen, which may provide a basis for homogenization of management among hospitals.

This study also has limitations: first, risk factors for chest compressions could not be further analyzed due to the small number of cases; second, as a clinical retrospective study, it is not possible to trace back the specifics of what was performed in resuscitation, and the quality of resuscitation could not be better assessed; and third, there may be a bias in the selection of samples due to the exclusion of the cases with admitted 24 h after birth and incomplete information.

## Conclusions

In conclusion, for VPIs, high-intensity DR resuscitation was associated with low GA and lower ANS utilization, and VPIs undergoing ETT resuscitation were at higher risk of adverse clinical outcomes. Therefore, enhanced perinatal and resuscitation management is of great significance to improve outcomes of preterm infants.

## Data Availability

The raw data supporting the conclusions of this article will be made available by the authors, without undue reservation.
